# Local Treatment of Typhoid Fever

**Published:** 1906-01-27

**Authors:** 


### Hospital Clinics.

### LOCAL TREATMENT OF TYPHOID FEVER.

In an interesting and comprehensive lecture on the treatment of typhoid fever Dr. William Ewart [1] advances some valuable suggestions in regard to the application of local measures to the intestinal lesions. Allowing that the typhoid ulcer is one of specific character, and that it therefore needs something more than mere local treatment, Dr. Ewart argues that there is abundant room for the application of topical measures comparable in principle to those adopted by surgeons in the treatment, say, of ulcer of the leg. Thus he argues that the indications are (1) to cleanse the ulcerated surface, (2) to keep it free from septic contact, (3) to protect the natural efforts towards repair, and (4) to promote healing by suitable stimulation of the local circulation. An initial cleansing is best secured by calomel or castor oil, or, better still, by the two in association. But to maintain the desired effect something more is needed, and thus he advises a frequent, sometimes daily, small dose of castor oil in the morning. This is called for, not merely with persistent constipation, but also where over-assiduous feeding leads to intestinal foulness and diarrhœa. Another measure by which the ulcerated and paralysed bowel may be saved from continuous maceration in foul warm juices is slight inclination of the bed, so as to evacuate and keep empty the cæcal cesspool. Complete asepsis of the bowel can hardly be hoped for, and probably none of the so-called intestinal antiseptics can be given in doses sufficient to ensure a perfect result. But short of this an approximation to the ideal can be obtained. Wedgewood's treatment by mercury and iron, or small six-hourly doses of calomel throughout the attack are valuable helps in this direction. It is desirable, further, not only to disinfect the intestine so far as possible, but also to reduce the bulk of the fæcal materials, and more especially of those which are prone to putrefactive changes—a consideration which obviously bears closely on the food supply permitted to the patient. Still, again, cleanliness and "protection" of the intestinal ulcers would be promoted were it possible to apply to them something comparable to the antiseptic agents employed in the dressing of external breaches of the surface. In the meantime it does not seem necessary to advance in this direction beyond the use of agents having for the most part mechanical and protective properties. Obviously for any such substance to reach the site of the lesion it must be neither soluble nor capable of absorption. Pure liquid paraffin meets these conditions and is in itself practically inert. Two teaspoonfuls every four hours is the maximum amount hitherto used by Dr. Ewart, but he suggests that this might be considerably increased. Another agent having disinfecting and deodorising power is vegetable charcoal in fine powder, and good results have been reported from doses so free as a tablespoonful every three hours. The view has also been advanced that charcoal prevents the absorption of toxins from the intestinal ulcers, and its action in promoting sleep in bad cases of typhoid fever has been explained in this way. Dr. Ewart has given charcoal suspended in water in doses of two teaspoonfuls every four hours, and notes only good effects from its employment. Any tendency to accumulation is prevented by the oily and laxative action of the liquid paraffin, and, as already explained, one or two drachms of castor oil in the morning, when the bowels fail to act at least once in the twenty-four hours, is an essential part of the treatment. Further, for the successful local action of the liquid paraffin and the charcoal on the intestinal ulcers it is obvious that an "empty" condition of the bowel must be cultivated. This presents the claim for the scheme of dietary to be so framed as to give "plenty of food" with "no fæces," which, in a word, means a skilled selection from the foods which leave "no residue"; the necessity for the presence of some insoluble material in the bowel being met by the administration of charcoal. For some years Dr. Ewart has been accustomed to use whey instead of milk for typhoid patients, and even whey may have to be diluted. Common salt (10 to 15 grains to the half pint) and sugar should be added to the whey, whilst other mineral and organic salts can be conveniently supplied in watery extracts, duly strained, of fruits and vegetables. The juices of various fruits, and vegetable soup or broth lightly flavoured with fresh beef or bacon and scrupulously clarified, are also grateful vegetable dishes, and fruit jellies, especially apple jelly, are in the same category. The nitrogenous elements are secured in peptonised whey, or in white of egg diffused through whey before peptonising. Eggs (in the absence of any adverse idiosyncrasy) are a valuable part of the dietary, and at a fairly early date even the yolk, in divided portions, may be given to the patient. Carbohydrates, as sugars, clarified honey, maltine, etc., are readily supplied in forms which, provided the supply of water for their solution in the intestine is sufficient, will leave no appreciable residue. Cream is the most acceptable form of fat and can be ordered up to an ounce a day. All the various foods above noted must be used with judgment and with appreciation of the demands of the individual case and of the several stages of the disease. Especially should it be remembered that it is a mistake to attempt much feeding during the first few days. At the outset even whey may yield more coagulum than the patient can digest. Thus two and a half pints of salted whey with plenty of water may be quite sufficient at the beginning, and later the whey may be fortified, and additions to the dietary be made on the lines already indicated. The absence or presence of distension of the abdomen and the state of the stools form the necessary guides to judgment in deciding whether to the basis of the food scheme—that is, salted whey—additions may or may not be made.

[1] Brit. Med. Jour., Dec. 9, 1905.

